# Positive Chest CT Features in Patients With COVID-19 Pneumonia and Negative Real-Time Polymerase Chain Reaction Test

**DOI:** 10.7759/cureus.9942

**Published:** 2020-08-22

**Authors:** Emre Pakdemirli, Uday Mandalia, Sherif Monib

**Affiliations:** 1 Radiology, West Hertfordshire Hospitals NHS Trust, Watford and St. Albans City Hospitals, London, GBR; 2 Radiology, West Hertfordshire Hospitals NHS Trust, Watford General Hospital, London, GBR; 3 General Surgery, West Hertfordshire Hospitals NHS Trust, Watford and St. Albans City Hospitals, London, GBR

**Keywords:** novel coronavirus, covid-19, 2019-ncov, sars-cov-2, chest ct, rt-pcr

## Abstract

Objectives

Clinically suspicious novel coronavirus (COVID-19) lung pneumonia can be observed typically on computed tomography (CT) chest scans even in patients with a negative real-time polymerase chain reaction (RT-PCR) test. The purpose of the study was to describe the CT imaging findings of five patients with negative RT-PCR results on initial and repeated testing but a high radiological suspicion of COVID-19 pneumonia.

Methods

Out of 19 clinically and/or radiologically diagnosed COVID-19 patients from our institution, five patients were selected for our study who had typical findings of COVID-19 on CT scan despite two negative RT-PCR results. Two district general hospital radiologists reviewed the chest CT images without prior knowledge of the RT-PCR test results. Scans were analyzed for the density of opacification and the distribution of disease.

Results

Out of 19 patients, five (26%) had initial negative RT-PCR test findings but positive CT chest features consistent with COVID-19. All patients had typical CT imaging findings of COVID-19. These included one patient with purely ground-glass opacities (GGO) and four patients with mixed GGO and consolidation. The typical distribution of parenchymal involvement was bilateral, posterior, and peripheral. Of the five patients with negative RT-PCR and positive CT findings, the range of CT severity score was 5 to 14. The median score, seen in three patients, was a score of 5, which corresponded to mild disease. One patient had a score of 8, corresponding to moderate disease, and one patient had severe disease with a score of 14.

Conclusion

Lung parenchymal changes related to COVID-19 can be seen on chest CT clearly despite repeated RT-PCR negative results.

## Introduction

The current novel coronavirus (2019-nCoV) pandemic was first identified in Wuhan, Hubei province, the Republic of China in December 2019 [1-4]. It was initially described as pneumonia of unknown origin but shortly afterward, the virus was identified as belonging to the coronavirus family and named novel coronavirus (COVID-19). From China, it spread rapidly across the globe, to be declared a pandemic by the World Health Organisation (WHO) on February 11, 2020 [5].

A chest X-ray is the primary imaging modality for investigating COVID suspected patients. The use of chest computed tomography (CT) as an imaging modality for patients with suspected COVID-19 is not well-established. From our cohort of 19 patients with typical CT findings for COVID-19, five patients had two negative reverse transcriptase-polymerase chain reaction (RT-PCR) results. 

RT-PCR tests for diagnosing COVID-19 harbors false-negative findings for a variety of reasons: the level of viral ribonucleic acid (RNA) being below detectable limits, insufficient cellular material for detection, and improper extraction of nucleic acid from clinical samples. These variables may explain why some COVID-19 tests are negative in the presence of apparent clinical disease.

In our report, we present the chest CT findings of five patients with COVID-19 pneumonia who had two consecutive, negative RT-PCR results. In this paper, we describe the clinical features and specific diagnostic imaging characteristics of COVID-19 for each patient.

## Materials and methods

This was a retrospective study for which the requirement for patients’ informed consent had been waived.

Patient data were collated from a hospital information encrypted safe site named Clinical Record Interactive Search (CRIS), picture archiving and communication system (PACS), patient web portal, patient discharge summaries, and NHS Trust encrypted web sites.

All data were anonymized and no patient contact was made.

Inclusion criteria for the study

**Figure 1:**
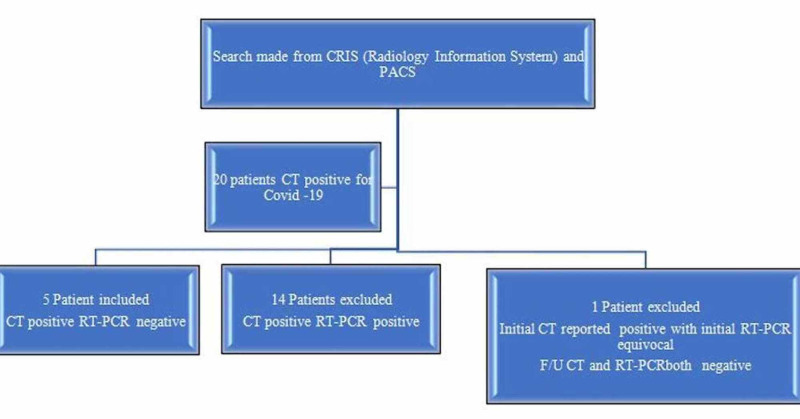
Patient selection flowchart

1) CRIS (Clinical Record Interactive system = Radiology Information System) and PACS searches were done from February 1 - May 24, 2020, for keywords “COVID”, “CT Chest”, “HRCT”, “COVID pneumonia”, “COVID-19”, “Novel Coronavirus”, Coronavirus pneumonia”, and “? COVID”.

2) Patients aged 18 to 100 years with symptoms of suspected COVID-19, investigated by chest CT and RT-PCR, between 1st February 1 to May 24, 2020.

3) Patients with a high clinical or initial radiological suspicion of COVID-19, e.g., fever, cough, shortness of breath (SOB), dependent on O_2_, and non-specific patchy shadowing on chest X-ray.

4) Incidental findings of COVID-19 on preoperative CT scan in otherwise asymptomatic patients.

5) Patients with RT-PCR and film array negative test results. Figure 1 shows the selection criteria.

Exclusion criteria

1) Patients younger than 18 years or older than 100 years

2) Patients with positive RT-PCR on initial or repeat testing

3) Patients with no radiological evidence of COVID-19

4) Patients with probable bacterial or other viral pneumonia (film array tests)

Having employed inclusion and exclusion criteria through careful selection, five of the 19 patients (3 women, 2 men; age range 42-53, mean age 48, median age 51) who received a chest CT were enrolled for the study. Fourteen patients were excluded from the study, as their initial and/or repeat RT-PCR results were positive. All patients underwent CT scanning of the chest on the same day that the initial RT-PCR test was performed.

CT protocols

Patients were scanned using either non-contrast high-resolution CT (HRCT), arterial phase chest CT, or CT pulmonary angiogram (CTPA). All images were obtained from a CT scanner (GE Frontier, GE Healthcare, Chicago, IL) with patients in the supine position.

The technical parameters for all examinations are summarized below:

HRCT and CT CAP: 120 kV, auto-modulated mA, 0.625 slice thickness, 512x512 matrix

CTPA: 100 kV, auto modulated mA, 0.625 slice thickness, 512x512 matrix

Image analysis

Images were reviewed by two UK-certified consultant radiologists (E.P. and U.M., with 25 and 5 years of experience, respectively). The chest CT images were reviewed individually by each radiologist on a picture archiving and communication system (PACS, Carestream Healthcare, Rochester, NY). Individual radiologists’ results were collated and discrepancies were reviewed together to achieve a consensus decision regarding severity score and lesion characteristics.

Chest CT images were evaluated on both mediastinal (width 350 HU, level 40 HU) and lung (width 1500 HU, level -500 HU) window settings. Two radiologists defined pulmonary lesions according to their location, density, shape, and margin.

The location of the lung lesions was recorded according to their lobar, axial, and anteroposterior distribution. The axial location of a lesion was classified as central (inner two-third of the lung) vs peripheral (outer one-third of the lung) and anterior vs posterior (defined by a dividing line drawn horizontally through the center of the chest). The densities of the lesions were classified as pure GGO, pure consolidation, or mixed.

Lesion shape was classified as amorphous, nodular, or wedge-shaped. Lesion margins were classified as well-defined or ill-defined. The presence or absence of the following 18 additional findings was also recorded: bronchial wall thickening, subpleural bands, vascular engorgement, halo sign, reverse halo sign, tree-in-bud, linear opacity, septal thickening, crazy paving, cavitation, cysts, pleural effusion, pleural thickening, pneumothorax, emphysema, mediastinal lymphadenopathy, subpleural sparing, and air bronchograms.

The vast majority of the lesions were patchy, with irregular, ill-defined margins. Therefore, lesion sizes were not assessed. Instead, a semi-quantitative severity score was employed for each lung lobe:

score 0 (0% involvement); score 1 (>0 - ≤25% involvement); score 2 (>25% - ≤50% involvement); score 3 (>50% - ≤75% involvement); score 4 (>75% - 100% involvement)

Visual severity scoring (from 0 to 4) was employed for each of the five lobes, giving a final cumulative severity score from 0 to 20 for each patient (Table 1).

**Table 1:** CT chest lesions’ morphological characteristics, locations, density, severity scoring, and other associated findings *n: number of patients; GGO: ground-glass pacification; CT: computed tomography

Predominant density	n* (total 5 patients)	Percentage (%)
Pure ground glass	-	0
Pure consolidation	-	0
GGO + Consolidation (mixed)	5	100
Laterality		
Bilateral	5	100
Unilateral	-	0
Predominant shape		
Amorphous	4	80
Rounded	1	20
Patchy	5	100
Pattern morphology and other associated findings		
Crazy paving	-	0
Mosaic pattern	-	0
Tree-in-bud	1	20
Halo sign	-	0
Reverse halo sign	-	0
Pleural thickening	1	20
Pleural effusion	1	20
Fibrosis	-	0
Mediastinal lymphadenopathy	1	20
Subpleural fibrotic line	3	60
Subpleural sparing	1	20
Focal vascular engorgement	2	40
Bronchial wall thickening	1	20
Septal thickening	3	60
Air bronchograms	-	0
Well-defined or ill-defined		
Well-defined	1	20
Ill-defined	3	60
Both	1	20
Lobar involvement (number of case bases)		
Right upper lobe	5	
Right middle lobe	5	
Right lower lobe	5	
Left upper lobe	4	
Left lower lobe	5	
Severity score (0-20)	5	
Mild (0 – 6)	3	
Moderate (7-13)	1	
Severe (14-20)	1	
Lesion distribution		
Central	1	
Peripheral	4	
No predilection	-	
Anterior	-	
Posterior	4	
No predilection	-	

Laboratory tests

All patients had nasopharyngeal swab tests for RT-PCR, film array, neutrophils, lymphocytes, C-reactive protein (CRP), and D-dimer tests.

## Results

A total of 19 patients were selected according to searches from the electronic patient records, CRIS, and PACS. Of these 19 patients, five (26%) had an initial negative RT-PCR test, but positive CT chest features consistent with COVID-19. Immediately after positive CT scan results, all patients were isolated for presumed COVID-19. Repeat RT-PCR swab tests were performed for three out of five patients. All 19 patients' film array tests were negative, therefore, the possibility of other bacterial and viral infections was excluded. In our cohort, 14 of the 19 patients (76%) were positive for both RT-PCR and chest CT findings and five of the 19 patients (study cohort) were negative RT-PCR and positive chest CT findings for COVID-19. 

Of the five patients with negative RT-PCR and positive CT findings at initial presentation, a severity score of 5 (mild) was seen in three patients, a severity score of 8 (moderate) was seen in one patient, and a severity score of 14 (severe) was seen in one patient.

All five patients had CT imaging findings typical of COVID-19, which included multifocal ground-glass opacity (GGO) (1 patient) and/or mixed GGO and consolidative changes (four patients). The distribution of lesions was also typical of COVID-19 infection with a bilateral, posterior, and peripheral predominance. 

Lesion pattern, morphology, and other associated findings are summarized in Table 1 while patient demographic features are described in Table 2.

**Table 2:** Patient demographics and brief clinical details

Number	Sex	Age	Clinical characteristics (Summary)
1	Female	42	Persistent cough and fever
2	Female	44	Persistent shortness of breath, pleuritic chest pain.
3	Male	53	Abdominal pain
4	Male	51	Abdominal pain, distension, and vomiting
5	Female	51	Asymptomatic, CT scan done as part of preoperative investigations

The detailed description of five patients with positive chest CT and negative RT-PCR testing are presented below:

Patient 1

**Figure 2:**
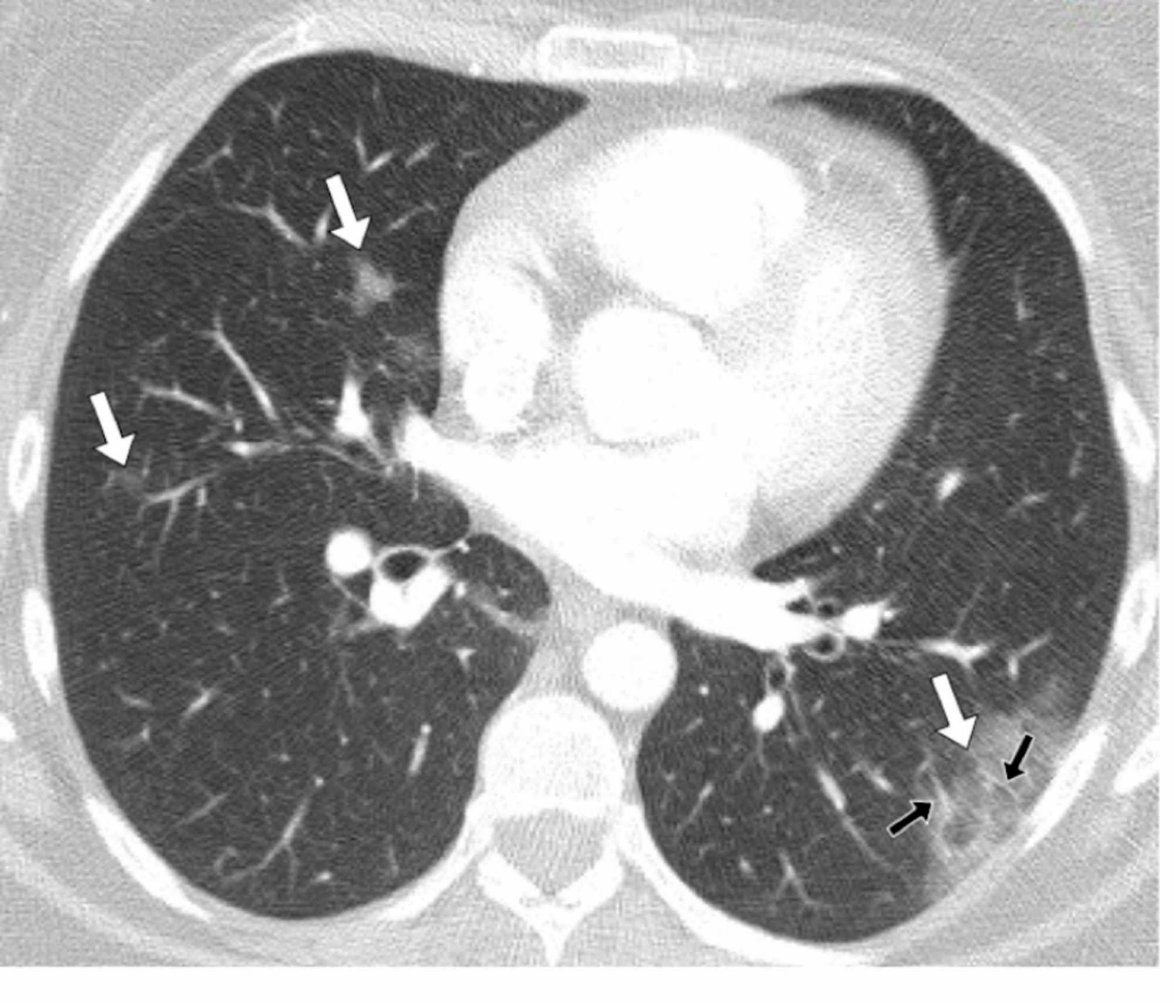
Patient 1 A 42-year-old female patient presented with a persistent cough and fever. Her D-dimer was 433 ng/mL and there was a clinical concern of a pulmonary embolus. A CT pulmonary angiogram (CTPA) scan was performed of the chest, which demonstrated changes consistent with COVID-19. A contrast-enhanced, axial CT image of the chest in lung windows showed bilateral patchy ground-glass opacification (white arrows). There was a large peripheral, posterior lesion in the left lower lobe in a distribution typical of COVID-19. There was also evidence of peripheral vascular engorgement (black arrows).

A 42-year-old female patient presented to the emergency department with fever, cough, and chest pain. There was initial clinical concern regarding either COVID-19 or community-acquired pneumonia as an underlying cause. The patient’s laboratory tests results included a normal white cell count, a high CRP of 86 mg/L (Normal: 0-5 mg/L), a high D-dimer of 433 ng/mL (normal range: 0-230 ng/mL), two negative RT-PCR and film array tests. In view of the raised D-dimer, the patient underwent a CTPA to exclude thromboembolic disease. Her chest CT showed multi-focal rounded GGO without parenchymal consolidation, predominantly involving subpleural peripheral regions of both lungs without anterior or posterior predilection (Figure 2). She was subsequently given a presumptive diagnosis of COVID-19 and her CT severity score was 5 (mild). No central or peripheral pulmonary embolus was detected. The patient was managed with simple analgesia, as it was felt that her chest pain was likely post-tussive. As her respiratory symptoms have been present for more than two weeks, it was felt that she no longer posed an infection risk and she was discharged.

Patient 2

**Figure 3:**
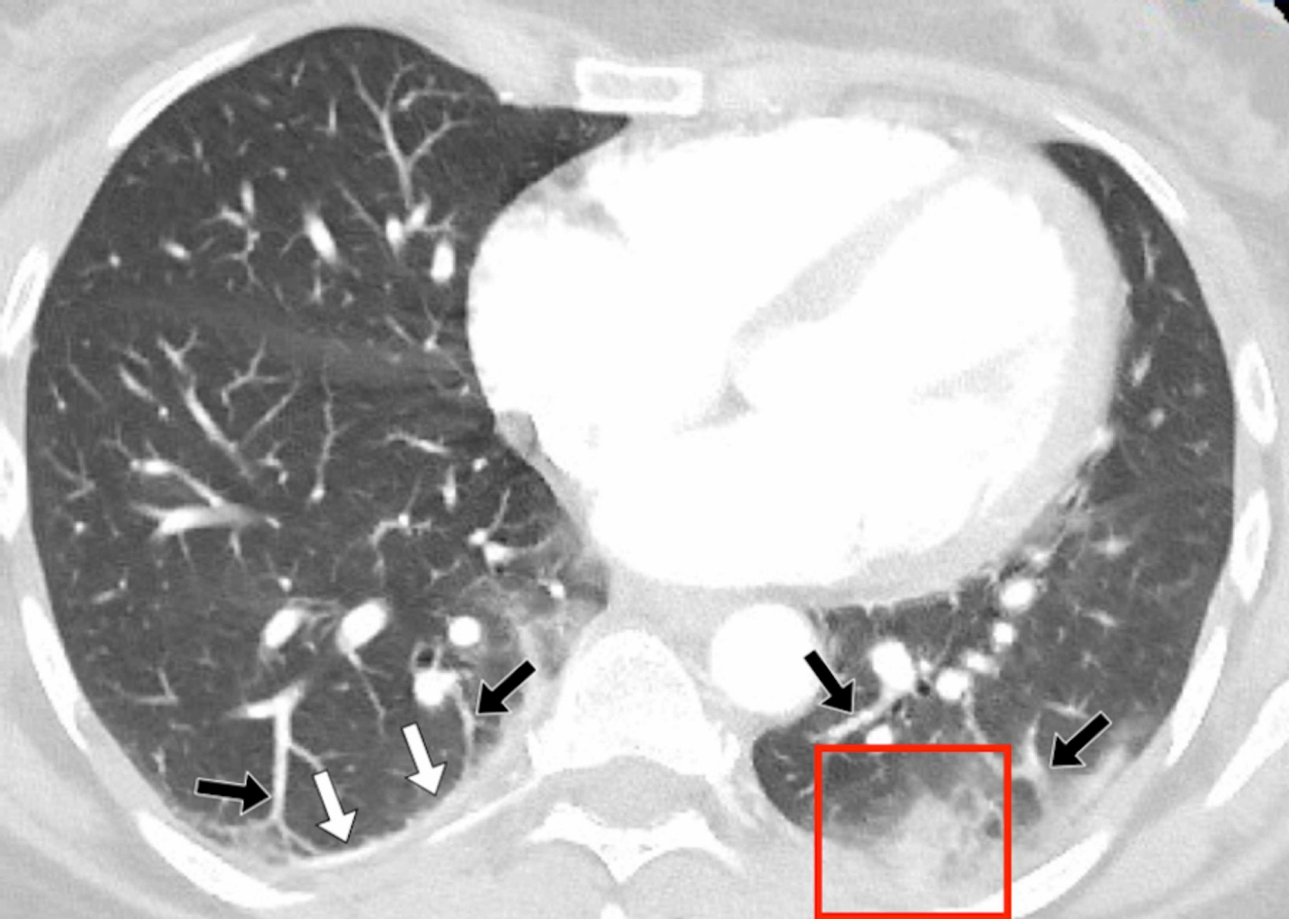
Patient 2 A 44-year-old female patient presented with persistent shortness of breath, pleuretic chest pain, and a raised D-dimer of more than 1400. A pulmonary embolus was suspected, and the patient was investigated with a CT pulmonary angiogram (CTPA). The CTPA showed no embolus; however, the patient did have features of COVID-19, which would account for her symptoms. Figure 3a demonstrates an axial, contrast-enhanced, CT image through the thorax, in lung windows. There was evidence of left basal consolidation (red box), right basal pleural thickening (white arrows), and basal vascular engorgement (black arrows).

**Figure 4:**
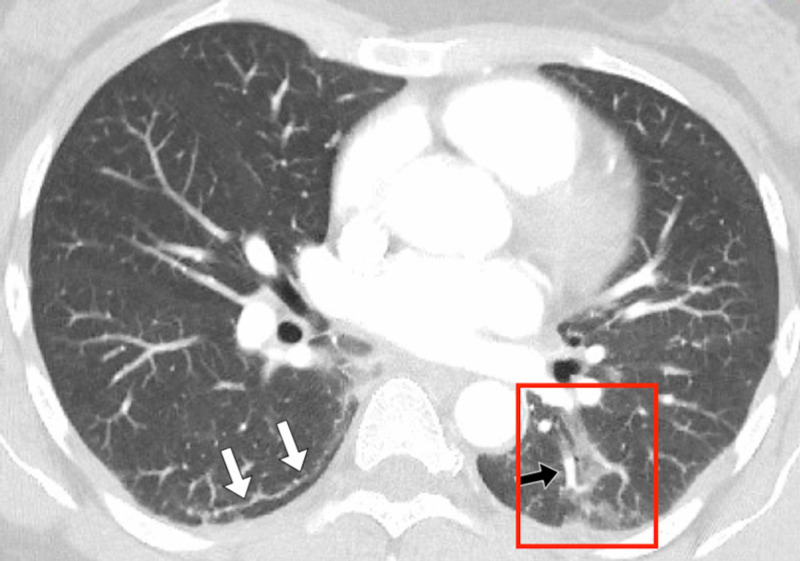
Patient 2 axial CT An axial computed tomography (CT) image in lung windows was taken at the level of the hila. The image shows a curvilinear subpleural reticular line (white arrows), vascular engorgement, and patchy peripheral and posterior ground glass opacification (red box).

A 44-year-old female patient admitted was admitted to the emergency department with persistent shortness of breath and pleuritic chest pain. Her laboratory findings revealed a high CRP of 9 mg/L and a high D-dimer of 1468 ng/mL. RT-PCR test was negative. Her chest X-ray prior to her CT scan showed bilateral lower zones patchy opacities. In view of her raised D-dimer and possible abnormalities on plain X-ray, a CTPA scan was performed on the same day. Her CT images showed patchy bilateral, mixed GGO and consolidative lesions, involving posterior and peripheral subpleural lungs (Figure 3, Figure 4).

Her CT severity score was 5 (mild). No central or peripheral pulmonary embolus was detected. As her symptoms and CT severity score were mild, she was discharged from the emergency department with standard COVID-19 isolation advice, without a second, repeat RT-PCR test, and no hospital follow-up was requested. 

Patient 3

A 53-year-old male patient was admitted to the emergency department with left-sided abdominal pain, decreased appetite, and vomiting. On physical examination, he described tenderness on the left side of the abdomen. The patient has mildly raised inflammatory markers with a CRP of 27 mg/L but no raised white cell count. A CT of the chest, abdomen, and pelvis was performed, which showed uncomplicated diverticulosis. Incidentally, images of the chest showed the typical changes of COVID-19. An RT-PCR swab test requested after the CT study was negative.

**Figure 5:**
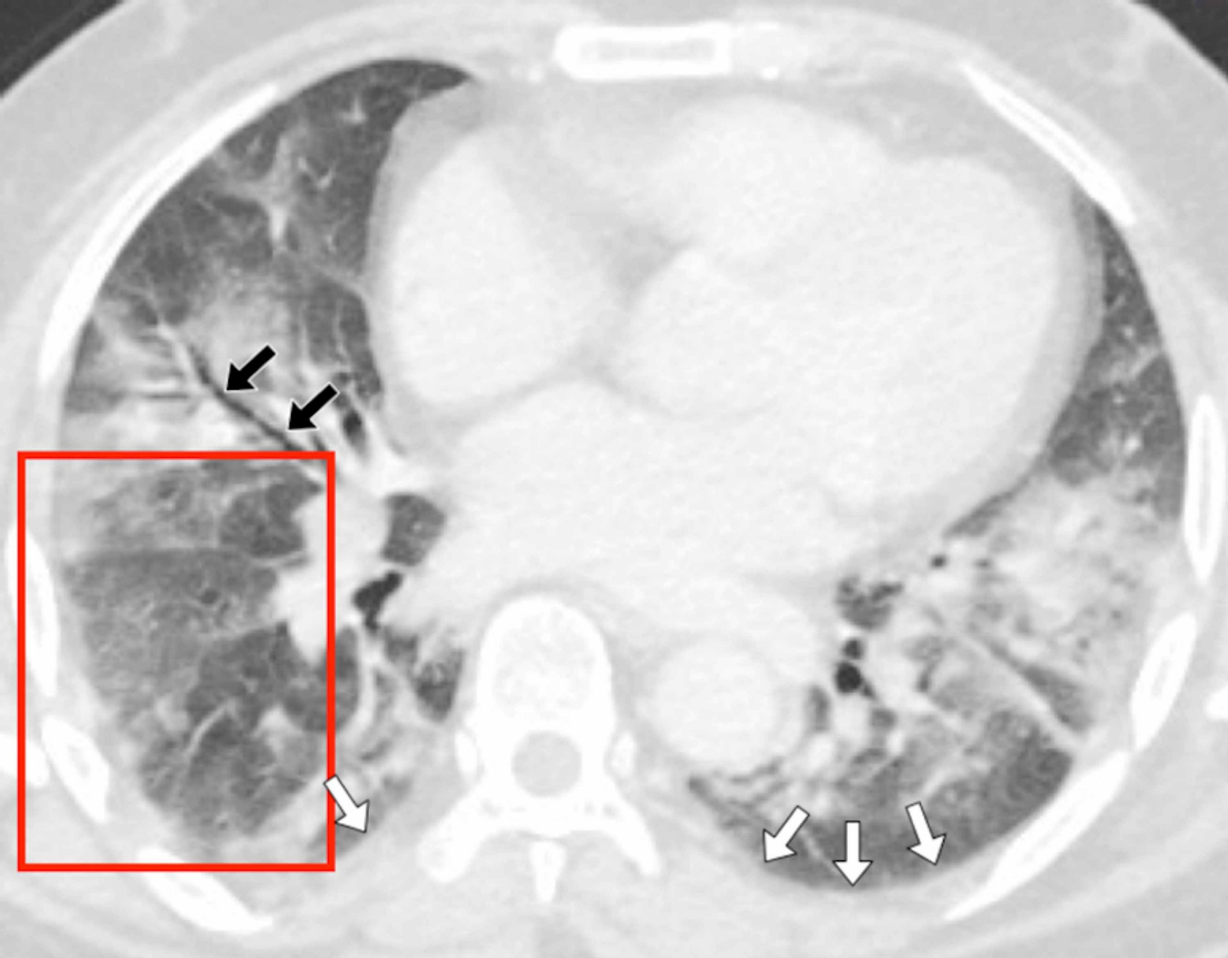
Patient 3 A 53-year-old male patient was admitted to the emergency department with left-sided abdominal pain, decreased appetite, and vomiting. The patient was investigated with a whole-body computed tomography (CT), which demonstrated lung parenchymal changes consistent with COVID-19. (Figure 5a) An axial contrast-enhanced CT of the chest, in lung windows, demonstrates patchy bilateral ground glass and consolidative changes. There is evidence of bi-basal pleural thickening (white arrows), air bronchograms (black arrows), and mosaic attenuation (red box).

**Figure 6:**
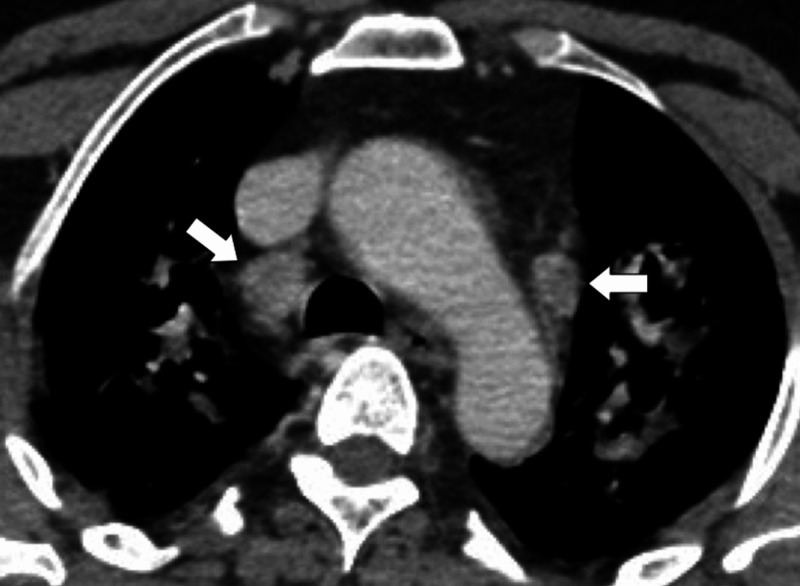
Patient 3 axial CT slice An axial computed tomography (CT) slice through the chest in the same patient, in mediastinal windows, demonstrates mediastinal lymphadenopathy (white arrows).

His CT images showed bilateral, amorphous, patchy areas of mixed GGO, and consolidative change involving the posterior and central lung fields (Figure 5, Figure 6). He also had mediastinal lymphadenopathy thought to be reactive. His CT severity score was 14 (severe). He was discharged from the surgical ward after two days of admission with standard COVID-19 isolation advice.

Patient 4

**Figure 7:**
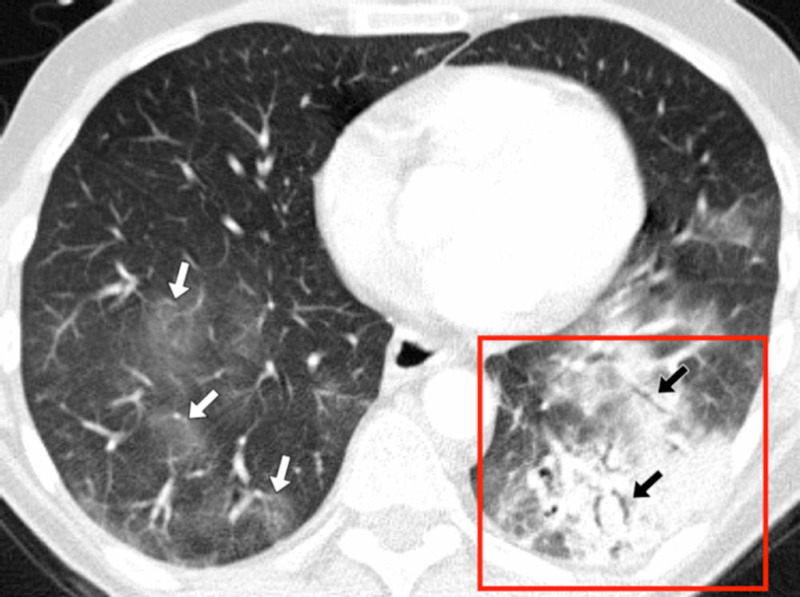
Patient 4 A 51-year-old male patient was admitted to the emergency department with a two-day history of vomiting. His abdomen was distended, with palpable bowel loops. He was investigated with whole-body computed tomography (CT). His abdominal CT (not shown) demonstrated small bowel obstruction, and imaging of the chest showed evidence of parenchymal changes consistent with COVID-19. Figure 4 shows an axial contrast-enhanced CT image of the chest, in lung windows. The image shows left basal consolidation (red box) with air bronchograms (white arrows). There are multiple, right, lower lobe ground glass-opacities (black arrows).

A 51-year-old male patient was admitted to the emergency department with a two-day history of vomiting. His abdomen was distended with palpable bowel loops. The patient's observation on admission revealed a low oxygen saturation of 94%, blood pressure of 120/70, a heart rate 120/min, a temperature of 37°C, and a respiratory rate of 17/min. Blood tests showed a raised CRP of 49.6 mg/L and a normal white cell count. An abdominal X-ray revealed dilated small bowel loops and a chest X-ray showed left lower lobe consolidation. There was no pleural effusions or pneumothorax. CT chest, abdomen, and pelvis confirmed the presence of small-bowel obstruction. This was thought to be related to adhesions from a previous appendicectomy. His chest CT images showed multi-focal patchy ground-glass opacities and parenchymal consolidation, with both ill- and well-defined opacities, predominantly involving the peripheral and posterior regions of both lungs; appearances typical of COVID-19 (Figure 7). He unusually also had a focal area of tree-in-bud opacification in the right lower lobe. His CT severity score was 8 (moderate).

He recovered with conservative treatment for bowel obstruction. He was isolated in the hospital and given supportive management with oxygen, intravenous (IV) fluids, and antibiotics. He had a catheter inserted prior to discharge for urinary retention and was followed up in the COVID-19 virtual clinic.

Patient 5

A 51-year-old female patient had been diagnosed with left breast cancer. She had finished her primary chemotherapy and was preparing for surgical treatment. On the day of her elective breast surgery, the patient underwent a chest CT examination as part of a pre-operative screen for COVID-19. She did not complain of any respiratory symptoms. Her subsequent laboratory findings were normal, including two negative RT-PCR tests.

**Figure 8:**
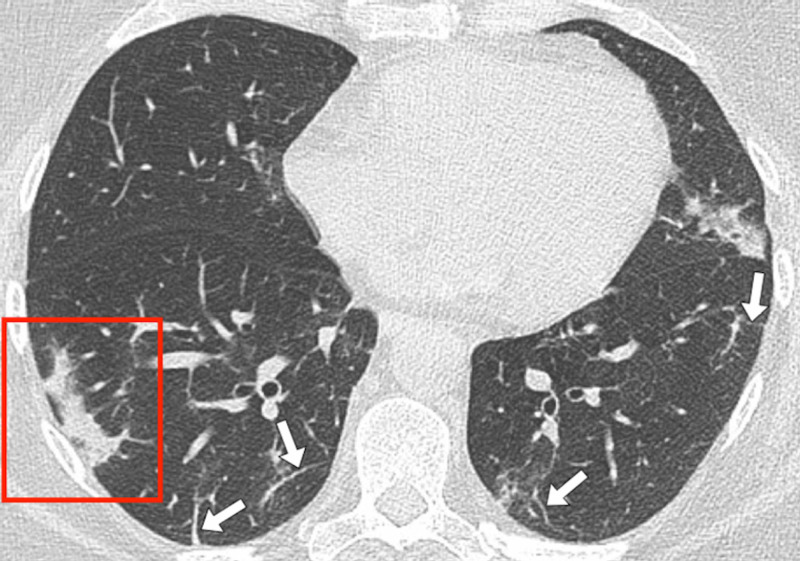
Patient 5 A 51-year-old female received staging computed tomography (CT) of the chest as part of her work-up for newly diagnosed breast cancer. She was asymptomatic with regard to respiratory symptoms. Her CT scan of the chest showed incidental changes consistent with COVID-19. Her subsequent real-time polymerase chain reaction (RT-PCR) swab tests were negative. Follow-up imaging with a chest X-ray showed the resolution of the consolidative changes. (Figure 8a) Axial contrast-enhanced CT slice of the thorax. There are bilateral patchy ground-glass and consolidative changes. There is a patch of linear consolidation seen in the periphery of the right lower lobe, a typical morphology for COVID-19 (red box). There are multiple thickened and irregular septa in both lung bases (white arrows).

**Figure 9:**
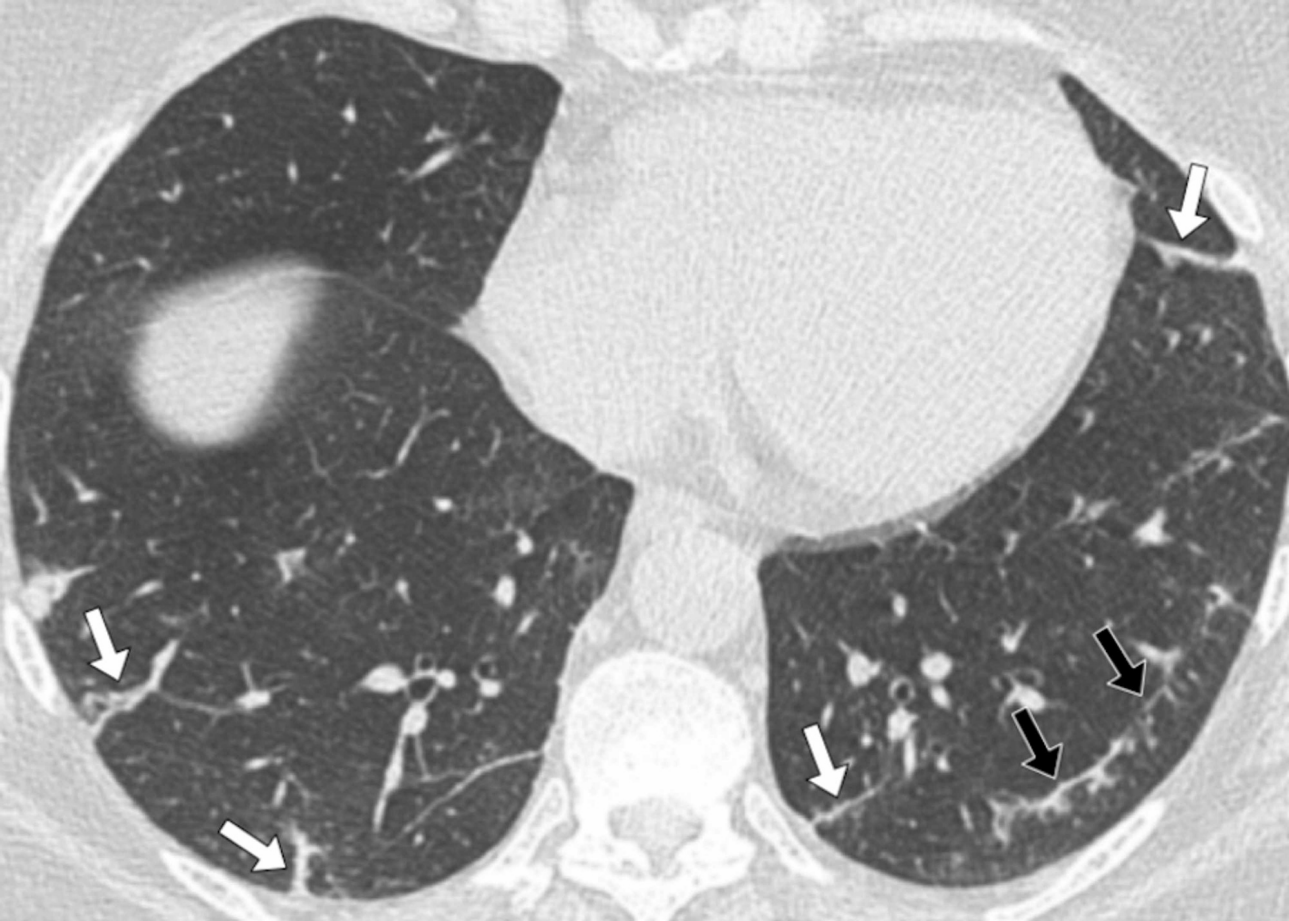
Patient 5 axial CT image An axial computed tomography (CT) image from the same study, at the level of the lung bases, demonstrates multiple thickened and irregular septae and a curvilinear subpleural line (black arrows). These features are commonly seen in COVID-19 patients.

Her chest CT revealed bilateral peripheral and posteriorly located mixed GGO and parenchymal consolidations with interlobular septal thickening (Figure 8, Figure 9).

The CT severity score was 5 (mild). The scheduled operation was canceled due to the incidental COVID-19 changes. The patient was discharged with advice to self-isolate. She was followed-up in three weeks with a repeat RT-PCR and chest X-ray. The subsequent chest X-ray showed significant regression of the consolidation with remnant peri-bronchial thickening and some residual interstitial changes.

## Discussion

Novel coronavirus COVID-19 is a new highly contagious viral disease, which has affected the whole globe. Diagnosis depends on the three cardinal clinical findings of fever, cough, and shortness of breath. The RT-PCR test has been regarded as a gold standard for the diagnosis of COVID-19 despite concerns regarding specificity and sensitivity. The test is time-consuming, and the availability of test kits has been a limiting factor with regard to monitoring the spread of disease. The chest CT manifestations of COVID-19 are being established but, to date, little is known about the disease course and treatment. X-ray is considered the first-line radiological tool for the diagnosis of COVID-19; however, CT is superior in disease recognition and follow-up. A radiological examination can be carried out well in advance of the RT-PCR test results and can, therefore, provide a role for patient triaging and surveillance.

Early recognition of the lung manifestation of the disease can also help initiate early supportive treatment and possibly prevent patients requiring intensive care where the disease outcomes are worse.

The review of these five cases has highlighted typical CT findings that may assist in the early detection of suspected cases and may help predict and prevent complications such as acute respiratory syndrome. Previous CT studies have shown that our cohort had typical features of COVID-19 on CT: a bilateral, multifocal, peripheral, lower zone, and posterior lung involvement [4,6-14]. We observed tree-in-bud [15] and mediastinal lymphadenopathy [16] in severe COVID-19 infection, which has not been frequently reported in the English literature. Crazy paving was not seen in our cohort, which can be seen in the late phase of the disease.

Limitations

There are several limitations to the study. The retrospective design and small cohort are the obvious limitations. Another limitation is the lack of resources, which restricted the number of repeat RT-PCR tests to not more than two. Out of our five patients, only three received two RT-PCR tests. Follow-up CT was not made, with the exception of a 51-year-old preoperative patient (Patient 5).

## Conclusions

CT scanning following chest X-ray plays an important role in detecting COVID-19 lung manifestations prior to the RT-PCT test results. COVID-19 can be diagnosed by CT prior to the RT-PCR test result, and RT-PCR test negativity should not exclude the diagnosis of this disease. In the context of the current pandemic, in patients with clinical symptoms or a history of exposure, CT features of viral pneumonia should be regarded as strongly suspicious for COVID-19 pneumonia, despite negative RT-PCR test results. We recommend that in these cases, patients should be managed with appropriate infection control measures and RT-PCR swab testing should be repeated.
